# Do They Already Feel Like Frauds? Exploring the Impostor Phenomenon in Children and Adolescents

**DOI:** 10.3390/children13010149

**Published:** 2026-01-21

**Authors:** Mona Leonhardt, Jane De Vries, Sonja Etzler, Sarah Peetz, Sonja Rohrmann

**Affiliations:** 1 Department of Psychology, Goethe-University Frankfurt, Theodor-W.-Adorno-Platz 6, Postfach 64 PEG, 60629 Frankfurt am Main, Germany; devries@psych.uni-frankfurt.de (J.D.V.); sonja.etzler@uniklinik-freiburg.de (S.E.); peetz@psych.uni-frankfurt.de (S.P.); rohrmann@psych.uni-frankfurt.de (S.R.); 2 Department of Psychosomatic Medicine and Psychotherapy, Medical Center, University Freiburg, Hauptstr. 8, 79102 Freiburg, Germany

**Keywords:** impostor phenomenon, personality traits, clinical symptoms, protective/risk factors

## Abstract

**Highlights:**

**What are the main findings?**

• Impostor feelings can already emerge in childhood and show a gradual increase across adolescence, indicating a developmentally sensitive trajectory rather than a phenomenon limited to adulthood.
• Higher impostor scores in children and adolescents are associated with a personality profile characterized by elevated neuroticism, lower extraversion, and consciousness, as well as lower self-esteem (level, stability, and contingency).

**What are the implications of the main findings?**

• The early emergence of impostor feelings underscores the necessity of conceptualizing impostorism as a developmentally salient, predominantly subclinical phenomenon with potential implications for mental health across the lifespan.
• Preventive efforts in childhood and adolescence, particularly those fostering stable self-worth and supportive, authoritative parenting, may help mitigate the consolidation of impostor feelings and related internalizing symptoms.

**Abstract:**

**Objectives:** The Impostor Phenomenon (IP), defined as persistent self-doubt despite objective success, has been extensively researched in adults. In contrast, empirical research on children and adolescents remains limited. **Methods:** The present study examines the prevalence, correlates, and potential risk as well as protective factors of the IP in a sample of 286 participants (56.6% female, 42.7% male, and 0.7% diverse) aged 8–18 years (*M* = 11.75, *SD* = 2.50). Participants were recruited from four distinct German subsamples between 2022 and 2024, including a clinically vulnerable group. The study employed a cross-sectional survey design administered to children and adolescents. **Results:** The results of the study indicate the presence of the IP as early as primary school age, with increasing intensity during adolescence. The study identified robust correlations between the IP and neuroticism, extraversion, conscientiousness, and self-esteem. In the present study, children and adolescents exhibiting depressive–anxious symptomatology demonstrated significantly elevated impostor scores in comparison to those manifesting other disorders. Furthermore, the study yielded negative associations between impostorism and various personal resources (e.g., optimism, self-efficacy) and family resources (e.g., parental support, authoritative parenting style). **Conclusions:** The present findings underline the importance of early intervention in addressing impostor feelings among younger age groups. In conclusion, the present findings contribute to our understanding of the IP etiology and underscore the importance of understanding the IP during formative years to inform prevention and intervention strategies.

## 1. Introduction

The impostor phenomenon (IP), first described by Clance and Imes [1], refers to an internal psychological experience in which individuals are unable to internalize their own successes despite objective indicators of excellent achievement. Instead of attributing their accomplishments to their own abilities, they trace their success back to external factors such as luck, chance, good connections, errors in assessment, or excessive effort. People with high impostor feelings doubt their abilities and are concerned that their environment might eventually recognize their alleged incompetence and expose them as frauds [2]. While most studies have focused on adults, the impostor phenomenon (IP) has only occasionally been investigated in children and adolescents [3,4]. Existing findings suggest that impostor feelings begin to develop and become evident already during childhood and adolescence. Thus, a thorough investigation of the IP across different developmental stages is crucial to understand its origins and trajectory.

The present study therefore aims to examine impostorism in younger populations and to investigate whether it is associated with constructs known to be relevant in adults, such as personality traits (e.g., neuroticism, low self-esteem). Moreover, the study aims to analyze underlying conditions as well as risk and protective factors, as familial background [5] and resources [6,7] also play a role in the development of the IP. Studies consistently report that individuals with pronounced impostor feelings experience higher levels of stress compared to those without such a self-concept [8]. Understanding the IP in younger age groups is crucial for informing the development of early preventive interventions. Children who show early signs of impostorism could benefit from targeted educational and therapeutic interventions aimed at mitigating the potential long-term effects like depression or anxiety [9,10].

### 1.1. Prevalence of Impostor Feelings in Children and Adolescents

The prevalence of pronounced impostor feelings in adults is estimated at around 40%, although figures vary considerably depending on the criteria applied [11,12]. The IP has been identified in various countries and cultures, including the USA [13], Iran [14], and Korea [15], and can therefore be considered as not culturally specific.

Findings regarding gender differences remain inconsistent: Several studies have found the IP to be unrelated to gender [7,16], while in some studies, women report higher impostor feelings than men (e.g., [17]), which aligns with the initial assumptions of Clance and Imes [1]. A recent meta-analysis [18] reported small to moderate effects, with women reporting slightly higher impostor feelings, particularly in academic settings. Furthermore, extant evidence suggests that these gender differences are somewhat less pronounced among professionals compared to postgraduates or undergraduates [12]. This trend may also be of relevance when examining the phenomenon of impostorism in children and adolescents. Further investigation is warranted into potential developmental and contextual factors that may influence these differences.

Age also appears to play a role, as impostorism in adults tends to decline across the lifespan [12]. Importantly, there is evidence that impostor feelings already occur in childhood [3], which corresponds to the developmental stage when self-concepts become more differentiated [19]. Socialization experiences, particularly parenting styles, have been discussed as relevant developmental factors, with responsive parenting identified as protective and high parental control as a risk factor [20,21]. These findings highlight that impostor feelings may originate during childhood and adolescence and may be shaped by contextual influences at an early stage.

With respect to age, a meta-analysis by Bravata et al. [12] focusing on adult populations revealed a negative correlation between age and impostorism, indicating that the IP tends to decrease with age but occurs across all age groups. Importantly, empirical studies have also demonstrated the occurrence of impostor feelings in children and adolescents. For instance, Chayer and Bouffard [3] demonstrated that even children in late primary school exhibit impostor feelings, albeit to a lesser extent. This finding is consistent with the broader observations concerning the development of self-concept in children [19]. Self-concept is defined as including self-referential knowledge about traits, competencies, interests, or habits. The development of this concept is influenced by a variety of sources, including family, peer groups, and educators. It is shaped by both positive and negative information, self-evaluations, and comparative assessments. As school-age children develop the capacity for self-reflection, their self-concept becomes increasingly differentiated, influenced by specific experiences in this developmental phase [19]. These patterns are consistent with the findings on the conditions under which the IP emerges.

It is widely accepted that the experiences encountered during childhood and adolescence, particularly those related to family socialization, play a crucial role in the development of IP [2,21]. Consequently, the role of socialization experiences, and more specifically the impact of parenting styles, has been identified as a significant developmental factor: Research has identified that responsive parenting behavior, characterized by sensitivity and a needs-oriented approach, functions positively [20]. Conversely, the presence of stringent parental oversight has been demonstrated to be concomitant with the emergence of impostor feelings [21]. The present findings emphasize the notion that impostor feelings may have their origins in early childhood and adolescence and that these feelings may be shaped by contextual influences during these formative stages.

### 1.2. Personality, Self-Esteem, and Impostorism

Empirical studies have demonstrated a positive correlation between impostorism and neuroticism and a negative correlation with extraversion. No significant correlations have been predominantly identified with agreeableness and openness [10,22]. Moreover, studies consistently report negative correlations between the IP and conscientiousness [10,22]. The finding, which initially appears contradictory, can be explained by the self-assessment tendencies of those affected. Individuals with higher impostor feelings are characterized by a tendency to underestimate their capabilities and achievements. It is hypothesized that the negative correlation with conscientiousness is attributable to this self-critical perspective, with affected individuals exhibiting overgeneralization of isolated behaviors perceived as indicative of a lack of conscientiousness to their general self-assessment [2]. This finding aligns with the observations reported by Vergauwe et al. [7], who demonstrated that individuals with higher levels in the IP tend to exhibit heightened self-criticism and set exceedingly high standards for themselves.

In addition, empirical research conducted on adult populations has repeatedly demonstrated a robust correlation between impostorism and diminished self-esteem (e.g., [10,23,24]). These findings have been replicated in different cultural contexts [12,15]. According to Rosenberg [25], high self-esteem is characterized by self-respect and an internalized sense of self-worth. Conversely, low self-esteem reflects a lack of self-respect and a perception of oneself as worthless, inadequate, or otherwise deficient [26]. Clance [27] posited that a lack of self-esteem constitutes a primary characteristic of the IP. A substantial body of impostor research suggests an involvement of self-esteem in the impostor phenomenon [8,12,28]. Moreover, differentiated evidence demonstrates the discriminant validity of the impostor phenomenon [10]. Therefore, the present study aims at investigating the relationship between the IP and self-esteem in children and adolescents. Beyond global self-esteem, research suggests that impostor feelings are related to self-esteem instability and to a stronger dependency of self-esteem on external validation and personal achievements [28,29]. It is plausible to expect similar associations between impostorism, personality, and self-esteem dimensions in children and adolescents, mirroring patterns observed in adults.

### 1.3. Clinical Perspectives

Impostor feelings are associated with substantial impairments in psychological well-being. In adults, higher levels of impostorism have been demonstrated to be consistently associated with elevated stress, depression, anxious tendencies, and social anxiety [5,9,10,13,30]. Yet, it remains unclear whether these associations already manifest in younger populations. Considering that adolescence is a critical period for the onset of internalizing disorders, examining the interplay between the IP and clinical symptomatology in youth is of particular importance. In the present study, we therefore included a clinical sample to investigate whether children and adolescents with depressive-anxious symptomatology report stronger impostor feelings than those with other mental disorders. This approach allows us to address whether the well-established links between impostorism and internalizing symptoms in adults are already evident in earlier developmental stages, thereby providing a clinically meaningful perspective on the IP.

### 1.4. Resilience Factors and Resources

In addition to risk factors, resilience factors have been identified as crucial in buffering against impostor feelings. In adult samples, self-compassion has emerged as a particularly relevant protective mechanism, as it promotes adaptive self-related attitudes and mitigates self-doubt [31]. These findings indicate that the availability of internal and external resources plays an important role in reducing the negative consequences of impostorism. However, research in this domain has so far focused on adults, leaving open the question of whether such protective mechanisms are already relevant at earlier developmental stages.

In addition to personal resources, social resources also appear to be highly relevant for understanding impostor feelings. Thus, parenting may be a central etiological factor. Feelings of inadequacy often arise because children fear that they will never live up to their parents’ expectations [32,33] and may exacerbate impostor feelings. Conversely, parental care is widely regarded as a crucial resource for children’s emotional and psychological stability [21,34]. Studies have demonstrated that parental care, as well as a responsive parenting style, is associated with impostorism [20,35], suggesting that parental warmth and support may serve as protective factors. Investigating personal and social resources in children and adolescents is therefore of particular importance, as impostor feelings may emerge early and subsequently solidify, increasing vulnerability to maladaptive outcomes later in life. Identifying resilience factors in younger populations can contribute to a better understanding of the developmental trajectories of impostorism and may inform early prevention strategies.

## 2. Method

### 2.1. Sample

In sum, 286 children and adolescents (56.6% female, 42.7% male and 0.7% diverse) aged between 8 and 18 years (*M* = 11.75, *SD* = 2.50) participated in the study. Data were collected between 2022 and 2024, thus covering a period following the most restrictive phases of the COVID-19 pandemic.

The study’s sample comprises four subsamples, as data collection was conducted in four recruitment phases. Participants were recruited through a children’s event at the university campus (sample 1: *n*_1_ = 81; *M*_1_ = 9.62, *SD*_1_ =1.65), messenger groups from school classes and clubs (sample 2: *n*_2_ = 89; *M*_2_ = 14.34, *SD*_2_ = 2.18), a clinical sample recruited from a child and adolescent psychotherapy practice during initial counseling/consultation sessions (sample 3: *n*_3_ = 21; *M*_3_ = 13.71, *SD*_3_ = 1.79), and a school project (sample 4: *n*_4_ = 95; *M*_4_ =10.75, *SD*_4_ = 0.65). Comparison of impostor scores between the non-clinical and clinical samples revealed a small, non-significant difference (*W* = 2961.5, *p* = 0.297, *d* = 0.24). Accordingly, and in line with the study’s primary focus on developmental associations, the samples were combined for the main analyses. Furthermore, for exploratory analyses within the clinical sample, participants were categorized based on the reported reason for presentation into two groups: (1) depressive–anxious symptomatology and (2) other symptomatologies.

### 2.2. Procedure

In all sub-samples, participation was voluntary, and participants were informed that they could withdraw from the study at any time without any negative consequences. Participation of the children required informed consent from their parents. Prior to the initiation of the study, ethical approval was obtained from the local ethics committee (2022-73b) and approval from the state-level educational authority of Hesse, Germany. (GWU 1331).

Following the provision of consent and preliminary sociodemographic data (e.g., age and gender), participants completed self-report questionnaires assessing the IP, personality traits, self-esteem, and risk as well as protective factors. In an effort to minimize the burden on the participants, only selected questionnaires were administered in each sub-sample. However, the Impostor Self-Concept Scale was included in all samples. Therefore, the duration of completion of the questionnaires ranged from approximately 20 min (e.g., Sample 1) to 45 min (Sample 4), contingent upon the number of questionnaires administered.

In Sample 1, children attending a children’s university event were approached together with their parents using an information poster over the course of four consecutive days. Following the provision of a concise verbal explanation, the children undertook the paper-and-pencil questionnaires autonomously and subsequently submitted them to the research personnel on site. In Sample 2, the recruitment for the online survey was facilitated through parent groups of school classes, clubs, and analogous settings via email or messenger services (e.g., WhatsApp). Participants or their parents were provided with a link to an online questionnaire administered via SoSci-Survey [36], accompanied by written study information. The survey was administered individually to children and adolescents. The third sample was recruited during initial consultation sessions at a child and adolescent psychotherapy practice. Questionnaires were administered to the participants in a paper-and-pencil format under the supervision of clinical staff. Participation or non-participation exerted no influence on the diagnostic or therapeutic process. In Sample 4, data collection took place within a school-based project involving four fifth-grade classes. Questionnaires were administered in a paper-and-pencil format during regular class time under teacher supervision. Across all samples, the participants were not provided with individualized feedback or financial compensation for their participation. However, they had the facility of receiving a certificate of participation. Nonetheless, the study’s participants and their parents were provided with debriefing information that was appropriate to their respective age groups. Furthermore, participants and other individuals involved in the study (e.g., parents and teachers) were offered the opportunity to attend an informational lecture on the IP. Contact information was also provided to address any further inquiries.

### 2.3. Questionnaires

Impostorism. IP was assessed with the Impostor Self-Concept Questionnaire for children and adolescents (ISF-KJ; [37,38]). The ISF-KJ contains 13 age-appropriate items (e.g., “Compared to my classmates, I often feel that others are more capable than I am.”) that are rated on a four-point Likert scale. In the present study, the internal consistency was α = 0.87.

Big Five. To assess the Big Five personality traits, the short version of the Big Five Inventory for Children and Adolescents (BFI-K KJ; [39]) was used. The BFI-K J contains 26 items that measure personality traits using a five-point Likert scale. Five items each measure the scales of neuroticism (e.g., “I am very worried.”) and agreeableness (e.g., “I easily trust others.”). Extraversion is assessed with three items (e.g., “I am sometimes shy and reserved.”) and openness to experience with seven items (e.g., “I am interested in many things.”). The degree of conscientiousness is measured using six items (e.g., “I complete tasks thoroughly.”). Internal consistencies ranged from α = 0.69 (agreeableness), α = 0.71 (conscientiousness), α = 0.71 (openness), and α = 0.75 (neuroticism) to α = 0.80 (extraversion).

Self-esteem. Self-esteem was measured using the Self-Esteem Inventory for Children and Adolescents (SEKJ; [40]), which assesses the level, stability and contingency of self-esteem using 32 items on a five-point scale. Ten items each measure self-esteem level (e.g., “I really like myself as I am.”) and self-esteem stability (e.g., “I generally like myself in the same way all the time.”). Twelve items assess the self-esteem contingency scale (e.g., “When I have difficulty understanding something, I do not feel less valuable because of it.”). The internal consistencies were α = 0.84 (self-esteem contingency), α = 0.85 (self-esteem stability), and α = 0.91 (self-esteem level).

Personal resources. The FRKJ 8-16 [41] measures six personal resources and four social resources in children and adolescents with 60 items on a four-point scale. To assess personal resources, six items each measure the scales of sense of self-worth (e.g., “I can be proud of myself.”), optimism (e.g., “I believe that everything will eventually turn out for the best.”), self-efficacy (e.g., “With my abilities, I can achieve anything.”), empathy and perspective-taking (e.g., “I can easily put myself in others’ shoes.”), sense of coherence (e.g., “I understand what is happening around me.”), and self-control (e.g., “I am good at waiting if I will receive something particularly great later.”). The internal consistencies ranged between α = 0.72 (self-control), α = 0.73 (sense of coherence), α = 0.83 (optimism and empathy), and α = 0.85 (self-efficacy), to α = 0.91 (sense of self-worth). To assess social resources, six items each measure the scales of parental emotional and social support (e.g., “When I feel down, my parents take care of me.”), authoritative parenting style (e.g., “My parents are warm, but also set boundaries for me.”), peer group integration (e.g., “I have friends I can rely on.”), and school integration (e.g., “I feel comfortable in my class.”). The internal consistencies span from α = 0.77 (peer group integration), α = 0.82 (authoritative parenting style), α = 0.87 (school integration), to α = 0.92 (parental emotional and social support).

### 2.4. Statistical Analyses

The statistical analyses were conducted using the open-source software R (Version 4.4.1; [42]) and the associated integrated development environment RStudio, (2024.12.0+467; [42]). The data that support the findings of this study are not openly available due to reasons of sensitivity and are available from the corresponding author on reasonable request. All participants (*N* = 286) who completed the survey were included in the following analyses. Scales with a maximum of 20% missing values were imputed with the median of the remaining items. For each scale, the total score was set as missing, if more than 20% missing item responses were recorded. In total, 0.42% of all values were imputed with the median. Before proceeding with the primary analysis, statistical assumptions for the analyses were examined. The requirements for all analyses were tested. Normality was not always met, but analyses were continued as these tests are robust against violations of normality (e.g., [43]). For the regression analyses, assumptions such as linearity, independence of residuals, homoscedasticity, and normality of error distributions were thoroughly assessed and confirmed. For each model, the Mahalanobis distance was calculated post hoc to identify multivariate outliers. Across all models, outliers were identified. In response to this finding, the models were reanalyzed with these outliers accounted for.

## 3. Results

**Figure 1:**
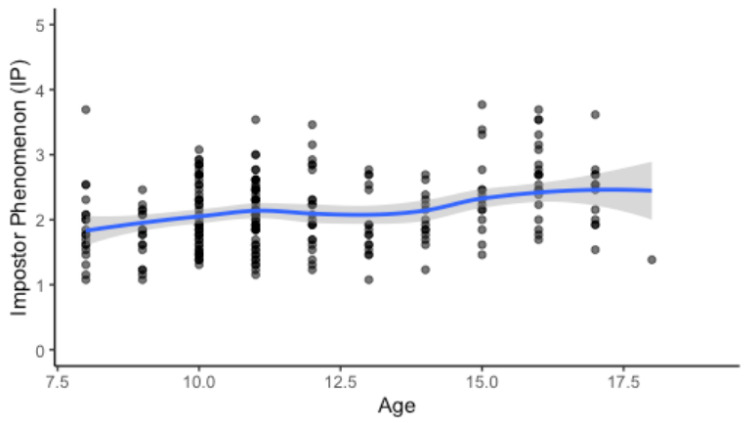
Impostor feelings across age (N = 236). Note. Higher values indicate stronger impostor feelings. Dots represent individual observations. The solid line depicts a LOESS smooth with 95% confidence interval (shaded area).

The mean sum score of the IP was *M* = 2.11 (*SD* = 0.59), indicating variability in the extent to which impostor tendencies were experienced in this age group. Regarding age, the findings indicated that impostor feelings can emerge in children as early as primary school. As demonstrated in Figure 1, impostor feelings were observed across the entire age range, including younger children. There was a slight but overall increasing trend in IP scores with increasing age. Descriptive analyses showed a statistically significant, small to moderate positive relationship between age and IP (*r* = 0.269; *p* < 0.001). Linear regression analysis further revealed that age significantly predicted IP scores, explaining 8.9% of the variance (*R*^2^ = 0.089, adj. *R*^2^ = 0.085), *F*(1, 236) = 22.95, *p* < 0.001. Age was thus identified as a significant predictor of impostor feelings (*b* = 0.067, *t* = 4.79, *p* < 0.001).

**Figure 2:**
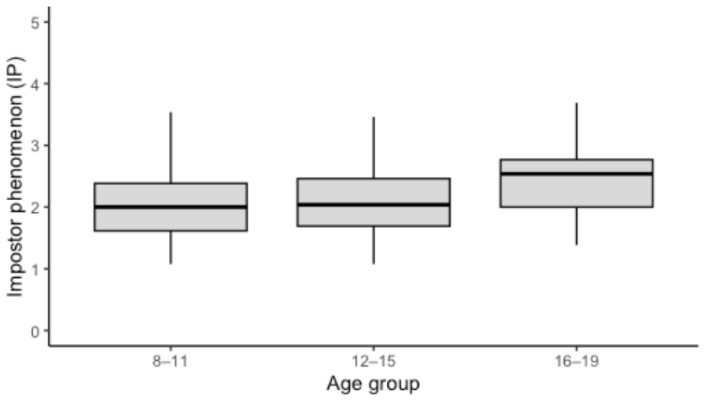
Impostor feelings across age groups (N = 236). Note. Higher values indicate stronger impostor feelings. Boxes represent the interquartile range (IQR), horizontal lines indicate the median, whiskers extend to the most extreme values within 1.5 × IQR. Age groups were defined as 8–11, 12–15, and 16–19 years.

Complementing these findings, age-group comparisons revealed a gradual increase in impostor feelings across developmental stages. As demonstrated in Figure 2, median IP scores were found to be lowest in children aged 8–11 years, increased slightly in early adolescence (12–15 years), and were highest in late adolescence (16–19 years). Mean IP scores increased from childhood (8–11 years: *M* = 2.03, *SD* = 0.52) to early adolescence (12–15 years: *M* = 2.14, *SD* = 0.58) and were highest in late adolescence (16–19 years: *M* = 2.53, *SD* = 0.61). A nonparametric Kruskal–Wallis test indicated significant differences between age groups, *H*(2) = 17.59, *p* < 0.001, with a moderate effect size (η^2^_h_ = 0.066). This finding suggests that impostor feelings increase across adolescence, with higher levels observed in older age groups.

The assessment of gender was categorized into three distinct classifications (female, male, and non-binary). Because only two participants identified as non-binary, thereby rendering this sub-group statistically non-viable, the analyses were focused solely on the female and male categories. Given the deviation of the male subsample from normality, a non-parametric test was employed to examine gender differences in IP. The Wilcoxon rank-sum test indicated that female participants reported significantly higher IP scores (*M* = 2.19, *SD* = 0.62) in comparison to male participants (*M* = 1.99, *SD* = 0.53), *W* = 7027.5, *p* = 0.007, *r* = 0.159, reflecting a small effect size [44].

In consideration of the developmental disparities between girls and boys during childhood and adolescence, the study incorporated age as a covariate to investigate the persistence of the observed gender disparity in impostor feelings beyond the confines of developmental effects. When both age and gender were entered into a regression model simultaneously, age remained a significant predictor of impostor feelings (*b* = 0.06, *p* < 0.001), while the effect of gender was reduced to a non-significant trend (*b* = −0.14, *p* = 0.066). This finding indicates that the previously observed gender disparity in impostor feelings may be predominantly attributable to developmental timing as opposed to gender per se.

### 3.1. Correlations Between IP, the Big Five Personality Traits, and Self-Esteem

As demonstrated in Table 1, IP scores showed a positive correlation with neuroticism and a negative correlation with extraversion and conscientiousness. No associations were found with openness or agreeableness. To further examine these relationships, we conducted separate regression analyses with IP as the dependent variable and each of the relevant Big Five dimensions as predictors. Neuroticism was found to be a significant positive predictor of IP scores (*b* = 0.436, *t* = 13.336, *p* < 0.001), accounting for 41.8% of the variance (*R*^2^ = 0.418; adj. *R*^2^ = 0.415; *F*(1, 248) = 177.8, *p* < 0.001). Extraversion emerged as a significant negative predictor (*b* = −0.248, *t* = −7.696, *p* < 0.001), explaining 19.53% of the variance (*R*^2^ = 0.199; adj. *R*^2^ = 0.195; *F*(1, 239) = 59.23, *p* < 0.001). Furthermore, conscientiousness also significantly negatively predicted IP scores (*b* = −0.218, *t* = −4.468, *p* < 0.001), with the model explaining 7.2% of the variance (*R*^2^ = 0.072; adj. *R*^2^ = 0.069; *F*(1, 254) = 19.98, *p* < 0.001).

As shown in Table 2, IP scores were negatively correlated with the self-esteem scales. To further examine these associations, we conducted a multiple regression analysis with IP as the dependent variable and self-esteem level, stability, and contingency as predictors. Together, the self-esteem variables explained 55.4% of the variance in IP scores (*R*^2^ = 0.554, adj. *R*^2^ = 0.546), representing a statistically significant model, *F*(3, 176) = 72.85, *p* < 0.001. All three facets of self-esteem emerged as significant negative predictors of IP scores: self-esteem level (*b* = −0.181, *t* = −4.079, *p* < 0.001), self-esteem stability (*b* = −0.158, *t* = −4.079, *p* = 0.001), and self-esteem contingency (*b* = −0.245, *t* = −5.635, *p* < 0.001).

In consideration of the previously observed age-related differences in impostor feelings, age was incorporated as an additional predictor to examine whether the associations between self-esteem and impostorism persisted beyond developmental effects. When age and the three self-esteem dimensions were entered simultaneously into the regression model, age was no longer a significant predictor of IP (*b* = −0.011, *p* = 0.458), whereas all self-esteem dimensions remained significant. The extended model accounted for 54.0% of the variance in IP scores (*R*^2^ = 0.540).

### 3.2. Clinical Sample Analyses

To examine potential group differences in impostor feelings, we first compared the clinical sample with the non-clinical participants. A Wilcoxon rank-sum test indicated no significant difference between the groups (*W* = 2961.5, *p* = 0.297). To further account for potential demographic confounds, we subsequently conducted an age- and gender-matched comparison between the clinical sample and a demographically comparable subsample of non-clinical participants. The matched analysis revealed no statistically significant disparities in impostor feelings (*W* = 236, *p* = 0.705), indicating that the absence of group differences was robust when demographic characteristics were taken into account. Within the clinical sample, we further explored whether impostor feelings varied depending on diagnostic symptom profiles. Children and adolescents with depressive–anxious symptomatology reported significantly higher impostor scores (*M* = 2.49, *SD* = 0.56) compared to those with other disorders (*M* = 1.74, *SD* = 0.33), *t*(19) = 3.299, *p* = 0.004).

### 3.3. Personal and Social Resources

As demonstrated in Table 3, IP scores were negatively associated with several personal and social resources. Specifically, higher IP scores were associated with lower self-worth (*r* = −0.321, *p* < 0.01) and optimism (*r* = −0.30, *p* < 0.01), as well as a weaker sense of coherence (*r* = −0.268, *p* < 0.01), and lower self-efficacy (*r* = −0.260, *p* < 0.05). Furthermore, the IP was negatively related to higher parental support (*r* = −0.243, *p* < 0.05) and an authoritative parenting style (*r* = −0.247, *p* < 0.05). No associations were observed with empathy (*r* = 0.024), self-control (*r* = −0.041), integration into the peer group (*r* = −0.172), and school integration (*r* = −0.204).

## 4. Discussion

The findings of the present study demonstrate that impostor feelings emerge during childhood, specifically at primary school age. Despite the modest effect sizes observed, the results suggest a developmental pattern in which impostor feelings emerge in early life and intensify during adolescence. Findings from correlational and regression analyses indicated a positive association between age and impostor feelings. This suggests that impostor experiences intensify as children transition into adolescence. Age-group comparisons further substantiated this pattern. The median and mean impostor scores were lowest among children of primary school age. These scores increased significantly during early and late adolescence. These results indicate that impostor feelings are not limited to adulthood but are also present in childhood and follow a gradual developmental trajectory. This finding is consistent with prior research indicating a lower manifestation of impostorism at younger ages [3] and an increasing intensity of impostor experiences with age [4]. The observed age effect is consistent with prevailing assumptions concerning the adolescent developmental phase, during which cognitive maturation, genetic influences, and environmental factors such as family, peers, and school contexts interact to shape personality traits and self-perceptions [45]. In particular, theories of self-concept development emphasize adolescence as a critical period for the differentiation and consolidation of self-concept [19]. During this phase, the salience of peer relationships and social evaluation may be increased, potentially amplifying self-doubt [19] and, consequently, fostering impostor feelings. Furthermore, it has been demonstrated that self-esteem often declines during puberty [46], which may contribute to a greater vulnerability to impostor feelings in adolescence. Concurrently, the present findings imply that age-related disparities in impostor feelings are not independent of broader self-evaluative processes. When self-esteem dimensions were included alongside age in regression analyses, the significance of age as a predictor of impostor feelings was no longer evident. However, all self-esteem facets remained robustly associated with impostorism. This pattern suggests that developmental increases in impostor feelings are predominantly attributable to individual differences in self-esteem, rather than to age per se. In summary, age appears to be a distal indicator of developmental change, while self-esteem represents a more proximal mechanism linking development to impostor experiences. From a developmental perspective, this interpretation helps situate the present findings within a broader lifespan framework. Studies have shown that self-esteem tends to improve with age in adulthood [47], and also impostor symptoms generally decrease later in life [12]. These findings indicate that impostor feelings observed in adolescence do not necessarily persist throughout the lifespan but rather reflect the developmental and social challenges characteristic of this period. Thus, while adolescence may represent a critical risk phase for the development and consolidation of impostor tendencies, adulthood may provide compensatory mechanisms, such as increased self-acceptance and greater stability of self-concept, that mitigate impostor experiences.

Initial gender analyses indicated that girls exhibited significantly higher impostor scores compared to boys, thereby supporting earlier assumptions that impostor feelings are more pronounced among women [1] and aligning with recent meta-analytic findings on adults [18]. However, when age was statistically controlled for, the gender effect was substantially attenuated and no longer statistically significant. This finding underscores the necessity of incorporating developmental timing considerations into the interpretation of gender disparities in impostor feelings. The onset of puberty and psychosocial development in girls typically occurs earlier than in boys. This phenomenon may, at least in part, account for the observed gender disparity. Consequently, the present findings imply that the previously documented gender disparities in impostor feelings may, at least in part, be indicative of developmental rather than gender-specific influences. Beyond developmental timing, the mechanisms underlying impostor feelings are also likely to differ between girls and boys. The gender-specific trajectories of self-esteem, with girls experiencing a stronger decline during adolescence [48], may partly account for higher impostor feelings in younger females. Furthermore, factors that may contribute to higher IP scores in girls likely differ from those in boys [49]. Previous research has shown that female college students exhibited stronger social comparison tendencies alongside higher IP scores [3]. However, factors like self-perception, social support, and social comparison were not examined, highlighting the need for future research.

In line with empirical studies in adults, our sample of children and adolescents showed predominantly the established associations between IP and the Big Five personality traits. As expected, elevated IP scores demonstrated a robust correlation with neuroticism and a negative association with conscientiousness and extraversion. Thus, these factors can be regarded as predisposing elements for the development of the IP. In consideration of the temporal stability of personality structure [50] and the close association between IP and personality, it can be deduced that a long-term tendency towards imposter feelings can be anticipated, along with sustained psychological strain for those affected. Further research is required, encompassing longitudinal studies that span the developmental phases of childhood, adolescence, and adulthood.

Regarding self-esteem, the findings indicated that self-esteem level, stability, and contingency emerged as significant negative predictors of IP scores. Thus, children and adolescents exhibiting lower, less stable, and less contingent self-esteem reported experiencing stronger impostor feelings. Importantly, these associations remained robust when age was included as a covariate, whereas age itself no longer significantly predicted impostor feelings. This aligns with previous research identifying low self-esteem as a core feature of impostor feelings [10]. The negative associations between these self-esteem dimensions and the IP are plausible given its inherent feelings of inadequacy, underestimation of achievements, fear of failure, and social comparison [51]. These findings emphasize the importance of fostering an autonomous, stable, and resilient sense of self-worth, for instance, through constructive feedback from caregivers.

Previous research linking the IP to parental rearing styles and family factors has predominantly relied on retrospective reports of adults concerning their childhood experiences (e.g., [5,21,30]). In contrast, the present study directly examines children and adolescents. The results of the present study provide further evidence to support the hypothesis that parenting styles have a significant impact on the development of impostor tendencies. Whilst social integration at school and within the peer group appeared to play a less prominent role in the data, significant associations were identified with parental support and an authoritative parenting style. Authoritative parenting combines high demands with high responsiveness, expressed through warmth and openness to communication [52], setting clear behavioral boundaries while simultaneously showing affection and support [21]. Children of authoritative parents are more likely to develop a stronger sense of social responsibility, independence, and self-control [53]. While the observed correlations with this parenting style were weak, they align with theoretical models and findings in adults, suggesting that a supportive social environment may serve as a protective factor against impostor feelings. With regard to personal resources, impostor scores demonstrated a negative correlation with core protective factors, including self-efficacy and optimism. It is noteworthy that no correlation was identified with empathy, suggesting that interpersonal sensitivity may not constitute a primary mechanism in impostor experiences during adolescence.

The incorporation of a clinical subsample enabled the extension of previous research on the relationship between impostorism and psychological well-being to younger populations. The analyses revealed no significant disparities in IP scores between clinical and non-clinical participants, which aligns with the concept of impostorism as a predominantly subclinical phenomenon [2]. Concurrently, the finding that impostor feelings are also reported within clinical populations underscores their relevance across both clinical and non-clinical contexts. Within the clinical sample, exploratory analyses indicated that impostor feelings were more pronounced among children and adolescents with depressive–anxious symptomatology compared to those with other disorders (e.g., attention deficits, tic disorders). This pattern is consistent with the well-documented associations between impostorism, depression, and anxiety in adults [12] and suggests that internalizing symptomatology may heighten vulnerability to impostor feelings already in youth. However, given the limited number of participants in the clinical subgroups, these findings should be interpreted with caution and considered as preliminary.

Taken together, these findings contribute to our understanding of the IP etiology by demonstrating that the interplay between parenting, personal resources, and impostor feelings can be observed concurrently in youth, rather than only through retrospective accounts in adulthood. However, there are some limitations that should be considered when interpreting the results. First, the utilization of self-report measures must be contemplated. Younger children, in particular, may experience difficulty in providing an objective assessment of their own abilities, characteristics, or behaviors. Second, the cross-sectional design of the study limits the ability to draw broad conclusions, as it does not track changes in the variables over time. In addition, although the inclusion of a clinical sub-sample represents a strength of the study, the number of participants within the clinical subgroups was relatively small. Consequently, findings related to the clinical sample should be interpreted with caution and considered preliminary. Larger clinical samples are needed to allow for more robust subgroup analyses and to increase the reliability of conclusions regarding clinical indicators of impostor feelings.

These limitations may restrict the generalizability of the present findings. Nevertheless, the study provides a first overview of impostor feelings in children and adolescents. It is recommended that future studies include larger samples in order to facilitate more robust analyses. Furthermore, it is recommended that the associations with constructs such as attributional style, perfectionism, and other potential risk or protective factors for developing IP should be examined. Future research should include larger and more diverse samples and employ longitudinal designs to more adequately capture developmental dynamics. Furthermore, it is recommended that associations with additional constructs, such as attributional style, perfectionism, and other potential risk or protective factors for the development of impostor feelings, be examined.

## 5. Conclusions

The present study provides further evidence that impostor feelings can already emerge during childhood and follow a systematic developmental pattern across childhood and adolescence. While age was found to be positively associated with impostor feelings, this relationship was largely explained by individual differences in self-esteem. The present findings indicate that developmental increases in impostor feelings are more strongly linked to changes in self-evaluative processes than to age per se. Furthermore, the results of the present study demonstrate a notable congruence between the personality profile of children and adolescents with elevated impostor feelings and that of adult samples, as reported in previous studies. This resemblance was observed across the Big Five traits and self-esteem dimensions. The observed convergence across developmental stages suggests that impostorism may potentially reflect an early-emerging and stable configuration of self-evaluative processes. From a developmental perspective, the early emergence of impostor feelings can lay the foundation for their potential persistence into adulthood, particularly when reinforced by individual vulnerabilities (e.g., low or unstable self-esteem) and contextual factors such as parenting styles. While impostor feelings were observed in both clinical and non-clinical groups, their heightened prevalence among youth with internalizing symptoms further supports conceptualizing impostorism as a subclinical yet psychologically meaningful phenomenon that may contribute to later mental health risk.

These results emphasize the need for early preventive efforts that foster a stable and resilient sense of self-worth in children and adolescents. Supportive parenting practices, constructive feedback, and developing self-efficacy may serve as protective factors against internalizing impostor beliefs. Future longitudinal research is essential to trace the developmental trajectory of impostor feelings across childhood, adolescence, and adulthood; identify causal mechanisms; and determine which personal and environmental resources may buffer their impact over time.

## Figures and Tables

**Table 1:** Descriptive statistics and intercorrelations of the Impostor Phenomenon and the Big Five personality dimensions (*N* = 246).

Variable	*M*	*SD*	1	2	3	4	5
1. Impostor Phenomenon	2.11	0.59					
2. Neuroticism	3.08	0.89	0.652 ***				
3. Extraversion	3.31	1.07	−0.428 ***	−0.412 ***			
4. Conscientiousness	3.25	0.75	−0.267 ***	−0.321 ***	0.155 *		
5. Openness	3.75	0.68	−0.010	0.004	0.153 *	0.272 ***	
6. Agreeableness	3.73	0.75	0.010	−0.016	0.106	0.243 **	0.394 ***

Note. The sample was composed of sub-samples 1, 2, 3 and 4. The Spearman rank correlation was used. *M* = mean, *SD* = standard deviation. * *p* < 0.05, ** *p* < 0.01, *** *p* < 0.001.

**Table 2:** Descriptive statistics and intercorrelations of the Impostor Phenomenon and Self-Esteem (*N* = 183).

Variable	*M*	*SD*	1	2	3
1. Impostor Phenomenon	2.11	0.59			
2. Self-esteem level	3.53	0.96	−0.57 ***		
3. Self-esteem stability	3.07	0	−0.611 ***	0.515 ***	
4. Self-esteem contingency	2.94	0.86	−0.656 ***	0.511 ***	0.578 ***

Note. The sample was composed of sub-samples 1, 2, 3 and 4. The Spearman rank correlation was used. *** *p* < 0.001. *M* = mean, *SD* = standard deviation.

**Table 3:** Descriptive statistics and intercorrelations of the Impostor Phenomenon and personal and social resources (*N* = 116).

Variable	*M*	*SD*	1	2	3	4	5	6	7	8	9	10
1. Impostor Phenomenon	2.11	0.59										
2. Self-worth	2.99	0.81	−0.321 **									
3. Optimism	2.78	0.63	−0.30 **	0.730 ***								
4. Self-efficacy	2.74	0.63	−0.260 *	0.722 **	0.691 ***							
5. Empathy	2.97	0.62	0.024	0.145	0.209 ***	0.293 **						
6. Sense of Coherence	2.90	0.56	−0.268 **	0.617 ***	0.662 ***	0.663 ***	0.348 ***					
7. self-control	2.73	0.62	−0.041	0.542 ***	0.418 ***	0.499 ***	0.312 **	0.454 ***				
8. Parental support	3.45	0.70	−0.243 *	0.484 ***	0.287 **	0.326 **	0.069	0.265 **	0.347 ***			
9. Authoritative parenting style	3.16	0.69	−0.247 *	0.324 **	0.141	0.269 **	0.288 **	0.268 **	0.434 ***	0.621 ***		
10. Integration Peer Group	3.13	0.61	−0.172	0.280 **	0.265 *	0.280 **	0.215 *	0.336 ***	0.281 **	0.385 ***	0.429 ***	
11. Integration School	2.83	0.69	−0.204	0.478 ***	0.385 ***	0.380 ***	0.209 *	0.330 **	0.502 ***	0.404 ***	0.356 ***	0.585 ***

Note. The sample was composed of sub-samples 1, 2, 3 and 4. The Spearman rank correlation was used. *M* = mean, *SD* = standard deviation. * *p* < 0.05, ** *p* < 0.01., *** *p* < 0.001.

## Data Availability

The data presented in this study are available on request from the corresponding author due to ethical and legal restrictions, as the dataset contains sensitive personal data from children and adolescents.
